# News and Notes

**Published:** 1903-05

**Authors:** 


### *NEWS AND NOTES.*

An epidemic of grip is reported at Yale University.

The island of Trinidad is suffering from an epidemic of small-pox.

The Marion Sims-Beaumont Medical College is reported to have been purchased by the St. Louis University.

The Medical College of Alabama at its commencement of April 3 graduated twelve in medicine and five in pharmacy.

The Medical Corps of the Navy is to be increased by 150, twenty-five of which are to be appointed each year for six years.

Surgeon-General Van Reypen states that there are twenty-seven vacancies in the grade of assistant surgeon in the United States navy.

The Fourth Pan-American Medical Congress, which was to have been held this year in Buenos Ayres, Argentine Republic, will not be held there.

The American Academy of Medicine is to hold its twenty-eighth annual meeting in the Arlington Hotel, Washington, D. C., May 11, 12, 1903.

The American Proctologic Society will hold its fifth annual meeting at the St. Charles Hotel, New Orleans, La., May 5 and 6. The program includes a meeting of the Executive Council at 1:30 p.m., May 5, followed by sessions at 2 o'clock, and again on Wednesday.

THE American Urological Association will hold its second annual meeting at the New Orleans Polyclinic, May 8 and 9, 1903. An interesting program has been announced.

---

THE offer of Andrew Carnegie to defray the expense of the new filtration plant at Cornell University, Ithaca, which will amount to about $150,000, has been accepted by the university.

---

THE New Orleans College of Pharmacy will hold its annual commencement exercises at the Atheneum, Wednesday, May 13, at 8 P.M. Hon. J. Y. Sanders will deliver the annual address.

---

THE National Conference of State Medical Examining and Licensing Boards will hold its thirteenth annual meeting in the common Council Chamber, City Hall, New Orleans, La., May 4, 1903.

---

DR. FLORENCE R. SABIN of Baltimore has been awarded the prize of $1000 offered two years ago for the best work of scientific research by women. Her communication was a study of the lymphatic system.

---

DR. JULIUS B. RANSOM, physician to the State prison at Dannemora, N. Y., says that one-fourth of the prisoners are tuberculous. There are accommodations for fifty-four out of the 250 consumptive prisoners.

---

DR. LOUIS HEISLER BALL, of Faulkland, Delaware, who was recently elected to the United States Senate from Delaware, is the second physician in active practice to become a member of the United States Senate.

---

MR. ANDREW CARNEGIE has offered to pay all the expenses of the Cornell students attacked by typhoid fever. The amount will reach, it is estimated, $50,000. At a recent-mass meeting of students the sum of $1000 was raised for this purpose.

---

WE take pleasure in making a correction of the error which appeared on page 40 of the April issue. The selection on Bright's Disease should have been credited to the *Southern California Practitioner*, instead of the source given.

---

THE Congress of American Physicians and Surgeons will hold its sixth triennial session May 12, 13 and 14, 1903, at Washington, D. C. One and one third railroad fare has been secured, to be purchased May 8-13 and good until May 16 returning.

---

PROF. KARL HOHN has invented a new musical instrument whose therapeutic virtues he expects to demonstrate at the World's Fair in St. Louis. The instrument is an addition to the armamentarium of alienists and "missionaries to the heathen." Its music puts the hearers to sleep. Its influence upon the performer is not described.

---

A LADY in Tennessee has sued the Pullman Company for $1999.99 for the loss of a night's sleep. She rode in a sleeping car from Nashville to Memphis. Among other occupants of the car were some members of the State legislature, who made a great deal of noise and subjected the lady to humiliation and annoyance, besides keeping her awake.

---

THE tests made by Dr. Hibbert Winslow Hills, bacteriologist of the Boston Board of Health, on the value of formalin injections to arrest bacterial activity in the body as advocated by Dr. Barrows, indicate that solutions to be efficient must be sufficiently strong to destroy tissues, and hence are impracticable.—*Journal of the American Medical Association.*

THE Mississippi State Medical Society met at Vicksburg April 15 to 17. This meeting was to take place at Greenville, but on account of high water there Vicksburg was selected. The following officers were elected: President, Dr. C. D. Mitchell; secretary, Dr. J. J. Haralson; treasurer, Dr. J. F. Hunter. Jackson was selected for the next meeting.

---

DR. HOWARD KELLY has brought suit against the Western Union Telegraph Co. for failure to deliver a telegram calling him to Cambridge, Md., to do an important operation. Dr. Kelly was on the through train from Boston to Baltimore, and telegrams were addressed to him at New Haven and at Trenton. Neither telegram was delivered, and the operation, one of urgent necessity, was not performed.

---

FROM Chicago comes the news of the death of a man only five feet ten inches high, yet weighing 480 pounds. It is said that he was so broad that he could not enter the turnstiles of the elevated railways in Chicago or the Illinois Central Railroad. The only surface cars in which he could ride were those having double sliding doors. To make him a complete suit of clothes required twelve yards of cloth, and underwear large enough for him was not manufactured in the United States. Everything he wore was therefore made to order in Europe. His hat was No. 12. The furniture provided for his use was made with special care, on account of his great weight.—*Exchange.*

---

THE twelfth annual convention of the Association of Military Surgeons of the United States will be held in Boston May 19 to 22 next. The president of the association, elected at the eleventh annual meeting at Washington last year, is Brigadier-General Robert A. Blood, surgeon-general. The committee of arrangements consists of Captain Miles Standish, Lieutenant-Colonel W. H. Devine, Lieutenant-Colonel Otis H. Marion, Major C. M. Green, and Lieutenant F. L. Gibson. Besides the official meetings, at which the governor will deliver an address, Dr. H. O. Marcy will give a luncheon at his residence. The secretary of the association is Major J. E. Pilcher, U. S. A.

---

THE American Congress of Tuberculosis for prevention of consumption announces the next meeting for St. Louis, Mo., U. S. A., July 18-23, 1904. The work of organization is being pushed as rapidly as possible. To facilitate this the congress has been granted a charter, thus making it a legal body, and by this means greatly facilitating the work of reorganization on the lines mapped out at the last meeting, when it was decided that a radical reorganization should be completed by the officers elected. For the purpose of completing the organization of the International, or World's Congress on Tuberculosis, on strictly ethical lines, a number of prominent physicians have been asked to serve on an Advisory Committee to assist the council in perfecting plans for the meeting. All have accepted and a large number will be added to the list.

---

### THE ETIOLOGY OF TUBAL PREGNANCY.

W. Hahn (*Münch. med. Woch.*, February 16, 1903) has given the subject of tubal pregnancy considerable study of recent years, and is of the opinion that gonorrhea is its most frequent cause, it being seen most often in large cities where gonorrhea is so prevalent. Hahn thinks the great increase of tubal pregnancy noted in recent years is due to the wide spread of gonorrhea and in part to the more exact methods of diagnosis and treatment. The condition is not an uncommon one, but is comparatively frequent. The prognosis is not to be regarded as fatal as was formerly believed, as the results of operative and non-operative cases show. The best prophylaxis against tubal pregnancy is the prevention of gonorrheal infection.—*Medical Age.*

